# Interactive Effects of CO_2_ Concentration and Water Regime on Stable Isotope Signatures, Nitrogen Assimilation and Growth in Sweet Pepper

**DOI:** 10.3389/fpls.2017.02180

**Published:** 2018-01-04

**Authors:** María D. Serret, Salima Yousfi, Rubén Vicente, María C. Piñero, Ginés Otálora-Alcón, Francisco M. del Amor, José L. Araus

**Affiliations:** ^1^Departament de Biologia Evolutiva, Ecologia i Ciències Ambientals, Universitat de Barcelona, Barcelona, Spain; ^2^Departamento de Hortofruticultura, Instituto Murciano de Investigación y Desarrollo Agrario y Alimentario, La Alberca-Murcia, Spain

**Keywords:** [CO_2_], nitrogen, sweet pepper, photosynthesis, water stress, δ^13^C, δ^15^N, δ^18^O

## Abstract

Sweet pepper is among the most widely cultivated horticultural crops in the Mediterranean basin, being frequently grown hydroponically under cover in combination with CO_2_ fertilization and water conditions ranging from optimal to suboptimal. The aim of this study is to develop a simple model, based on the analysis of plant stable isotopes in their natural abundance, gas exchange traits and N concentration, to assess sweet pepper growth. Plants were grown in a growth chamber for near 6 weeks. Two [CO_2_] (400 and 800 μmol mol^−1^), three water regimes (control and mild and moderate water stress) and four genotypes were assayed. For each combination of genotype, [CO_2_] and water regime five plants were evaluated. Water stress applied caused significant decreases in water potential, net assimilation, stomatal conductance, intercellular to atmospheric [CO_2_], and significant increases in water use efficiency, leaf chlorophyll content and carbon isotope composition, while the relative water content, the osmotic potential and the content of anthocyanins did change not under stress compared to control conditions support this statement. Nevertheless, water regime affects plant growth via nitrogen assimilation, which is associated with the transpiration stream, particularly at high [CO_2_], while the lower N concentration caused by rising [CO_2_] is not associated with stomatal closure. The stable isotope composition of carbon, oxygen, and nitrogen (δ^13^C, δ^18^O, and δ^15^N) in plant matter are affected not only by water regime but also by rising [CO_2_]. Thus, δ^18^O increased probably as response to decreases in transpiration, while the increase in δ^15^N may reflect not only a lower stomatal conductance but a higher nitrogen demand in leaves or shifts in nitrogen metabolism associated with decreases in photorespiration. The way that δ^13^C explains differences in plant growth across water regimes within a given [CO_2_], seems to be mediated through its direct relationship with N accumulation in leaves. The changes in the profile and amount of amino acids caused by water stress and high [CO_2_] support this conclusion. However, the results do not support the use of δ^18^O as an indicator of the effect of water regime on plant growth.

## Introduction

After tomato, sweet pepper is the second largest horticultural product cultivated in the Mediterranean basin in terms of area (del Amor, 2007). Moreover, a significant area is devoted to its cultivation under cover (greenhouses of different categories), and is frequently combined with the application of CO_2_ fertilization (Piñero et al., 2016). Essentially, a high CO_2_ concentration ([CO_2_]) stimulates photosynthesis and may contribute to increasing productivity in greenhouses while palliating environmental problems such as water stress or high temperature. Nevertheless, plant responses to elevated [CO_2_] may be affected in one or another way as a result of photosynthetic acclimation (Long et al., 2004) or due to differences in growing conditions such as water regime (O'Leary et al., 2015). Thus, some degree of water stress may increase the efficiency of water use, and at the same time positively affect the quality of the final product (López-Marín et al., 2017). However, the interaction of elevated [CO_2_] with water stress has not been widely studied and there are studies supporting either the positive or the negative effects of elevated [CO_2_] on water stress tolerance (Medina et al., 2016). Exposure to elevated [CO_2_] may mitigate the inhibition of photosynthesis under water stress and improve water use efficiency by a positive synergistic effect of both factors on stomatal closure, but can also stimulate oxidative stress and not affect plant growth (Erice et al., 2007, 2014; Bencze et al., 2014; Medina et al., 2016). In addition, plant size may limit the direct physiological effects of elevated [CO_2_] (Xu et al., 2016). Moreover, some studies indicated that this interaction is highly dependent on the genotypic variability and the severity of water stress (Erice et al., 2014; Medina et al., 2016; Xu et al., 2016).

Therefore, a better understanding of the interactions between high [CO_2_] and water stress is important for predicting the agricultural consequences of the expected increase in [CO_2_]. In the case of sweet pepper the genotypic performance and the specific responses to the combinations of [CO_2_] and water conditions have usually been monitored through photosynthetic and transpirative gas exchange traits (Peñuelas et al., 1995; del Amor et al., 2010). However, the use of methodologies that are able to integrate physiological processes on a larger temporal scale is an alternative that needs exploration. The analysis of the natural abundances of stable isotopes in plant matter may represent an alternative (Dawson et al., 2002; Araus et al., 2008) that is worth exploring.

Carbon isotope composition (δ^13^C), frequently expressed as discrimination (Δ^13^C) against the surrounding air, provides information on the effect of growing conditions on photosynthetic carbon assimilation (Farquhar et al., 1982; Condon et al., 1990). Plants discriminate against the heavier carbon isotope (^13^C) during photosynthesis and the extent of this discrimination depends on the ratio of the intercellular vs. the atmospheric [CO_2_] (C_i_/C_a_) in photosynthetic organs (Farquhar et al., 1982, 1989). Since the C_i_/C_a_ ratio depends on the balance between the photosynthetic activity and the stomatal conductance (A/g_s_) of the plant (Farquhar et al., 1982, 1989; Rebetzke et al., 2002; Condon et al., 2004), when analyzed in plant dry matter Δ^13^C becomes a time-integrated indicator of the A/g_s_ ratio and therefore of water use efficiency of the plant (Farquhar and Richards, 1984). Under drought stress, the Δ^13^C (or δ^13^C) is also a good predictor of stomatal conductance (Condon et al., 2002) and of water input received by the crop (Araus et al., 2003). For growing conditions where the water regime is not the main environmental variable, it remains challenging to assess whether variation in the carbon isotope signature is the result of changes in intrinsic photosynthetic capacity or stomatal conductance (Scheidegger et al., 2000; Farquhar et al., 2007). Due to this, the analysis of the stable oxygen isotope signature in plants may contribute toward understanding the nature of the changes in δ^13^C (Barbour and Farquhar, 2000).

The oxygen isotope composition (δ^18^O) in plant tissues can be influenced by three main factors. The first factor is the isotopic composition of the source water taken up by the plant (Roden and Ehleringer, 1999). The second factor is the enrichment in ^18^O in the leaves due to evaporation in comparison to source water (Pande et al., 1995). The third factor is the fractionation of oxygen isotopes during biochemical reactions involved in the synthesis of organic matter (Farquhar and Lloyd, 1993). Sugars and other metabolites formed in leaves incorporate the leaf water isotopic signal, which is then retained in structural organic compounds, such as cellulose (Barbour, 2007; Gessler et al., 2014). Stomatal conductance plays a crucial role in regulating the water balance of the plant. Providing there is constancy in the δ^18^O of the water taken up by the plant, the δ^18^O of plant matter integrates evaporative conditions throughout the life cycle of the plant, and this is largely unaffected by photosynthesis (Barbour and Farquhar, 2000; Farquhar et al., 2007). Therefore, δ^18^O has been proposed for estimating stomatal conductance and transpiration and thus plant water use in different species (Barbour and Farquhar, 2000; Barbour et al., 2000; Sheshshayee et al., 2005; Farquhar et al., 2007; Cabrera-Bosquet et al., 2009a, 2011; Cernusak et al., 2009a).

Nitrogen is the most growth-limiting nutrient element for plants (Piñero et al., 2016). In fact, the nitrogen isotope composition (δ^15^N) in plant matter is an indicator of the effect of growing conditions on the nitrogen metabolism of the plant, even though a complete knowledge of the underlying biochemical mechanisms is lacking (Cernusak et al., 2009b; Tcherkez, 2010; Yousfi et al., 2012). The natural abundance of δ^15^N has been used in sweet pepper to assess the source of nitrogen fertilization (Flores et al., 2007; del Amor and Navarro, 2008). However, to the best of our knowledge, studies on the interactive effect of [CO_2_] and water regime on the δ^15^N and δ^18^O of the plant are scarce.

This study compared the δ^13^C, δ^18^O, and δ^15^N, together with N concentration, of the leaf dry matter of sweet pepper plants growing under different [CO_2_] and water regimes. Further the stable isotope signatures of these three elements were correlated with plant growth. Moreover, gas exchange and amino acid profiles were measured on similar leaves. The final aim of this study is to produce a single conceptual model, integrating different key physiological traits, that explains the variability in sweet pepper biomass due to growing conditions and genotypic variability. To that end, plants were grown under hydroponic conditions at a relatively low light intensity and mild to moderate water stress, resembling the growing conditions usually experienced, in a mild Mediterranean climate, by sweet pepper plants within commercial plastic/polycarbonate greenhouses adapted to CO_2_ fertilization and which often include a shadow sheet (Dueck et al., 2006; del Amor and Gómez-López, 2009; del Amor et al., 2010; Pérez-Jiménez et al., 2016).

## Materials and methods

### Plant material and growth conditions

The experiment was carried at the Instituto Murciano de Investigación y Desarrollo Agrario y Alimentario (IMIDA), La Alberca, Murcia, Spain. Four sweet pepper (*Capsicum annuum* L.) cultivars were studied: Tallante (Ta; De Ruiter Vegetable Seeds, Inc.), Coyote (Co; Syngenta Seeds SA), Herminio (He; Syngenta Seeds SA), and Velez (Ve; Enza Zaden BV). These cultivars are commonly used in commercial greenhouses in SE Spain (Almeria and Murcia regions). Seedlings were transplanted to 5-l black containers filled with coconut coir fiber (Pelemix, Alhama de Murcia, Murcia, Spain) and acclimated during 5 days to the new conditions. Then three irrigation treatments were applied for 41 days: the control (an amount of 500 ml of nutrient solution was applied every day), mild stress (same amount every 2 days), and moderate stress (same amount every 3 days). Plants were irrigated with a modified Hoagland solution with the following composition in meq L^−1^; ${\text{NO}}_{3}^{-}$: 12.0; H_2_${\text{PO}}_{4}^{-}$: 1.0; ${\text{SO}}_{4}^{2-}$: 3.5; K^+^: 7.0; Ca^2+^: 4.5; Mg^2+^: 2.0. Irrigation was supplied via pressure-compensating and anti-drain drippers (2 l h^−1^) and fresh nutrient solution was applied to avoid salt accumulation, with a minimum of 35% drainage (del Amor and Gómez-López, 2009).

Plants were grown in a climate chamber designed to mimic usual environmental conditions experienced by pepper plants within greenhouses (del Amor et al., 2010), with fully-controlled environmental parameters: 70% RH, 16/8 h day/night photoperiod, 28/16°C and a photosynthetically-active radiation (PAR) of 250 μmol m^−2^ s^−1^ provided by a combination of fluorescent lamps (TL-D Master reflex 830 and 840, Philips, the Netherlands) and high-pressure sodium lamps (Son-T Agro, Philips, the Netherlands). Plants were grown at [CO_2_] of 400 μmol mol^−1^ (atmospheric [CO_2_]) and 800 μmol mol^−1^ (high [CO_2_]). The [CO_2_] was regulated by injection of external compressed CO_2_ (bottle [CO_2_] ≥ 99.9%), controlled by an infrared gas analyser (Dräger Politron IR CO_2_, Sweden). For each cultivar within a specific water regime and [CO_2_], five replications (each consisting in a single plant) were run. Thus, 24 treatments were studied, corresponding to the combination of four cultivars, three irrigation levels, and two [CO_2_], totalling 120 plants. All the study was done in the same growth chamber. Therefore, the experiment was done consecutively, with the only difference being [CO_2_]. We carefully verified that the germination and seedling growth conditions were the same (by using a small growth chamber (BINDER KBWF 240, BINDER GmbH, Tuttlingen, Germany), with light, temperature, and RH control. Plants were grown under a randomized complete block design (*n* = 60).

### Photosynthetic and transpirative gas exchange and chlorophyll content

At the end of the experimental period, net CO_2_ assimilation, stomatal conductance, transpiration, and the C_i_/C_a_ ratio were measured in the youngest fully-expanded leaf of five plants per treatment, using a CIRAS-2 portable photosynthesis system (PP Systems, Amesbury, MA, USA) with a PLC6 (U) Automatic Universal Leaf Cuvette of 1.7 cm^2^. The cuvette provided light (LED) with a photon flux of 800 μmol m^−2^ s^−1^, 400 or 800 μmol mol^−1^ [CO_2_] and a leaf temperature of 22°C. Water use efficiency (WUE) was determined as the ratio of net CO_2_ assimilation to transpiration.

The leaf chlorophyll content on an area basis was determined in the same leaves used for gas exchange with a SPAD-502 (Konica-Minolta Sensing, Japan) portable meter. Three measurements were made on each leaf.

### Measurement of tissue anthocyanins

Anthocyanins were extracted from oven-dried (after a minimum of 72 h at 65°C), ground tissue samples of plant leaves, suspended in acidified methanol (methanol:water:HCl, 79:20:1, by vol.), autoextracted at 0°C for 72 h, centrifuged and absorbance measured at 530 and 657 nm for each supernatant (Mirecki and Teramura, 1984). Anthocyanin concentration was calculated as Ab_530_ nm-1/3 Ab_657_ nm g^−1^ dry matter (Lindoo and Caldwell, 1978).

### Leaf water and osmotic potentials and relative water content

The leaf water potential (Ψ_w_) was measured in the same leaves used for gas exchange determinations, using a Scholander pressure chamber (model 3000, Soil Moisture Equipment Corp., Santa Barbara, USA) as reported elsewhere (Turner, 1988). Measurements were performed after gas exchange determinations and then the leaves were put in Eppendorf tubes with holes at the bottom and rapidly frozen. These tubes were then centrifuged twice, at 4,000 g for 4 min (4°C), using an Eppendorf centrifuge so that all sap was extracted from the samples. The osmotic potential (Ψπ) of the leaf sap was assessed with a vapor pressure osmometer (Wescor 5500, Logan, Utah, USA) used to measure the osmolality (mmol kg^−1^) of the expressed sap; this was converted to osmotic potential according to the Van't Hoff equation: Ψπ (MPa) = −RTC, where R is the gas constant (0.00832 l MPa K^−1^ mol^−1^), T is the temperature (293 K) and C is the number of moles of solute in 1 kg of water (= 1 l at 293 K). The leaf relative water content (RWC) was measured on same-age leaves as those used for Ψπ. Three small disks (2.07 cm^2^) per leaf from each of the six plants were cut and weighed immediately to obtain fresh mass (FM), and then they were placed for 24 h in the dark in a beaker (30 cm^3^) filled with distilled water. After this, they were reweighed to obtain turgid fresh mass (TM), and dry mass (DM) after drying at 80°C for 48 h. The relative water content (RWC), expressed as a percentage, was calculated as RWC = [(FM–DM)/(TM–DM)] × 100%.

### Shoot biomass

Plants were harvested at the end of the experiment (41 days after transplanting), and 120 plants (five plants per treatment) were analyzed. The aerial parts (thereafter referred as shoot biomass, including leaves plus stems and petioles) were dried and the dry weight determined after a minimum of 72 h at 65°C. The specific leaf area (SLA) was calculated as the ratio between the area and the dry weight of leaf discs of 6.91 cm^2^.

### Leaf N-total concentration and stable carbon, nitrogen, and oxygen isotope composition

Total nitrogen concentration and the stable carbon (^13^C/^12^C) and (^15^N/^14^N) isotope ratios in the whole pool of shoot leaves were measured using an elemental analyser (Flash 1112 EA, ThermoFinnigan, Germany) coupled with an isotope ratio mass spectrometer (Delta C IRMS, ThermoFinnigan, Germany), operating in continuous mode. Samples of 1 mg and reference materials were weighed into tin capsules, sealed, and then loaded into an automatic sampler (ThermoFinnigan, Germany) prior to EA-IRMS analysis. Measurements were carried out at the CCiT (Centres Científics i Tecnològics) of the University of Barcelona. Nitrogen was expressed as a concentration (percent of dry weight). The ^13^C/^12^C ratios were expressed in δ notation (Coplen, 2008): δ^13^C (‰) = (^13^C/^12^C)_sample_/(^13^C/^12^C)_standard_−1, where “sample” refers to plant material and “standard” to international secondary standards of known ^13^C/^12^C ratios (IAEA CH7 polyethylene foil, IAEA CH6 sucrose, and USGS 40 L-glutamic acid) calibrated against Vienna Pee Dee Belemnite calcium carbonate (VPDB) with an analytical precision (*SD*) of 0.10‰. The same δ notation was used for the ^15^N/^14^N ratio (δ^15^N), but in this case using international secondary standards of known ^15^N/^14^N ratios (IAEA N_1_ and IAEA N_2_ ammonium sulfate and IAEA NO_3_ potassium nitrate) referred to N_2_ in air, with an analytical precision of 0.18‰.

For the δ^18^O, the ^18^O/^16^O was determined by an on-line pyrolysis technique using a Thermo-Chemical Elemental Analyser (TC/EA Thermo Quest Finnigan, Germany) coupled with an IRMS (Delta C Finnigan MAT, Germany). Samples of 1 mg were weighed into silver capsules, sealed and oven-dried at 60°C for not less than 72 h to remove moisture and loaded into an automatic sampler. Results were expressed as δ^18^O values, using two secondary standards (IAEA 601 and IAEA 602) calibrated against Vienna Standard Mean Oceanic Water (VSMOW) (Coplen, 2011); the analytical precision was ≈ 0.25%. Analyses were conducted at Iso-Analytical Limited (Crewe, Cheshire, UK).

### Free amino acids

The free amino acids were extracted from leaves (frozen at −80°C): the sap was extracted, after vortexing at 5,000 rpm (10 min, 4°C), and analyzed by the AccQ·Tag-ultra ultraperformance liquid chromatography (UPLC) method (Waters, UPLC Amino Acid Analysis Solution, 2006). For derivatization, 70 μl of borate buffer was added to 10 μl of the fruit sap and 20 μl of reagent solution. The reaction mixture was mixed instantly and heated at 55°C for 10 min. After the temperature was lowered, an aliquot of the reaction mixture was used for injection. The column was an Acquity BEH C18 1.7 μm, 2.1–100 mm (Waters), and the wavelengths were set at 266 nm (excitation) and 473 nm (emission). The solvent system consisted of two eluents: (A) AccQ·Tag-ultra eluent A concentrate (5%, v/v) and water (95%, v/v); and (B) AccQ·Tag-ultra eluent B. The following elution gradient procedure was used for the analysis: 0–0.54 min, 99.9% A−0.1% B; 5.74 min, 90.9% A− 9.1% B; 7.74 min, 78.8% A−21.2% B; 8.04 min, 40.4% A−59.6% B; 8.05–8.64 min, 10% A−90% B; 8.73–10 min, 99.9% A−0.1% B. The injection volume was 1 μl, with a flow rate of 0.7 ml min^−1^. The temperature of the column was maintained at 55°C. External standards (Thermo Scientific) were used for the quantification of the amino acids, and Empower 2 (Waters) software for data acquisition and processing.

### Statistical analysis

Data were subjected to factorial ANOVA to test the effects of the growing conditions ([CO_2_] and water regime), genotype, and their interactions. Mean comparisons were performed using Tukey's honestly significant difference (HSD) test. A bivariate correlation procedure was used to calculate the Pearson correlation coefficients between the different traits measured. Multiple linear regression (stepwise) analysis was used to analyse the criterion included to explain variation in shoot biomass under different growing conditions. Principal component analysis was produced to analyse the interrelationships among the shoot biomass, leaf nitrogen concentration, and chlorophyll content, the stable isotope composition of C, O, and N, and the gas exchange variables. Data were analyzed using IBM SPSS Statistics 24 (SPSS Inc., Chicago, IL, USA). Figures were created using a Sigma-Plot 11.0 program for Windows (Systat Software Inc., Point Richmond, CA, USA). Finally, we performed path analyses (Li, 1975) to quantify the relative contributions of direct and indirect effects of stable isotopes and other key traits on aboveground biomass. This methodology offers the possibility of building associations between variables that is based on prior knowledge. A path analysis determines simple correlations between independent factors (in this case δ^13^C), and regresses them on each intermediary (*C*_*i*_*/C*_*a*_, g_s_, δ^15^N and N concentration) or dependent factor (shoot biomass) to obtain direct effects in the form of partial regression coefficients (i.e., path coefficients). This model was aimed at understanding biomass responses to genotypic differences across water regimes under different levels of [CO_2_]. A model with a comparative fit index (CFI) (Arbuckle, 1997) with values > 0.9 was taken as indicative of a good fit. Data were analyzed using the Amos Graphics package (IBM SPSS Amos, USA). A clustered heat map of amino acid profile was built using the R statistics environment (R Development Core Team, Vienna, Austria). Additionally, after generating a correlation matrix of all parameters analyzed in R, we performed a network analysis of significant correlations (Pearson correlation coefficient cut-off of 0.7 and *P* < 0.05) under ambient and elevated [CO_2_] using Cytoscape software (Shannon et al., 2003).

## Results

### Effect of growing conditions and genotype on shoot biomass

Compared with control plants water stress negatively affected shoot biomass (SB), plant height (PH), and the leaf water potential (Ψ_w_), whereas leaf chlorophyll (LC) content slightly increased and no differences existed in the specific leaf area (SLA), relative water content (RWC), leaf osmotic potential (Ψπ), and anthocyanin content (Table 1). Increasing ambient [CO_2_] significantly increased SB, PH, LC, anthocyanin, and Ψπ, whereas all the other traits reported in Table 1 decreased. A genotypic effect was significant for all the traits except RWC, anthocyanin, and Ψπ.

**Table 1:** Water regime, CO_2_ concentration and genotype effects on biomass, growth parameters, anthocyanin content, and leaf water status of four sweet pepper genotypes grown under different combinations of CO_2_ concentration and water supply.

	**SB**	**PH**	**SLA**	**LC**	**anthocyanin**	**RWC**	**Ψ_w_**	**Ψπ**
**WATER REGIME**								
Control	10.48^a^ ± 0.18	39.80^a^ ± 0.59	946.58^a^ ± 22.50	44.12^b^ ± 0.46	0.17^a^ ± 0.03	85.88^a^ ± 0.74	−3.88^c^ ± 0.18	−7.17 ^a^ ± 0.16
Mild water stress	8.65^b^ ± 0.19	36.40 ^b^ ± 0.61	920.83^ab^ ± 23.24	46.20^a^ ± 0.47	0.24^a^ ± 0.04	85.04^a^ ± 0.77	−5.18^b^ ± 0.18	−6.87^a^ ± 0.16
Moderate water stress	6.35^c^ ± 0.20	35.09 ^b^ ± 0.64	860.20^b^ ± 25.54	45.71^a^ ± 0.52	0.18^a^ ± 0.02	85.87^a^ ± 0.85	−6.52^a^ ± 0.20	−6.98^a^ ± 0.18
**CO_2_ CONCENTRATION**								
400 ppm	7.78 ± 0.14	35.90 ± 0.46	996.30 ± 17.63	43.89 ± 0.36	0.13 ± 0.01	88.70 ± 0.58	−4.90 ± 0.14	−7.72 ± 0.12
800 ppm	9.52 ± 0.17	38.88 ± 0.55	822.62 ± 21.07	47.15 ± 0.43	0.27 ± 0.03	81.87 ± 0.70	−5.49 ± 0.16	−6.21 ± 0.15
**GENOTYPES**								
Coyote	7.93^d^ ± 0.21	37.30^b^ ± 0.67	919.52^ab^ ± 25.66	46.32^ab^ ± 0.52	0.22^a^ ± 0.04	85.53^a^ ± 0.85	−5.32^ab^ ± 0.20	−7.02^a^ ± 0.18
Herminio	8.76^bc^ ± 0.22	40.20^a^ ± 0.70	867.48^b^ ± 26.92	46.87^a^ ± 0.55	0.21^a^ ± 0.03	86.48^a^ ± 0.89	−5.21^ab^ ± 0.21	−7.10^a^ ± 0.19
Tallante	8.38^cd^ ± 0.22	34.27^c^ ± 0.72	965.42^a^ ± 27.36	43.36^c^ ± 0.56	0.16^a^ ± 0.05	85.96^a^ ± 0.91	−5.40^a^ ± 0.21	−7.03^a^ ± 0.19
Velez	9.31^a^ ± 0.24	37.05^b^ ± 0.78	886.92^ab^ ± 29.79	44.66^bc^ ± 0.61	0.19^a^ ± 0.03	84.40^a^ ± 0.99	−4.60 ^b^ ± 0.23	−6.89^a^ ± 0.21
**LEVEL OF SIGNIFICANCE**								
Water regime (WR)	214.01^***^	455.48^***^	38058^ns^	60.31^**^	0.15^ns^	0.95^ns^	76.42^***^	2.54^ns^
CO_2_ concentration (CO_2_)	52.15^***^	153.01^***^	521286^***^	183.80^***^	0.32^***^	806.83^***^	5.86^**^	49.06^***^
Genotype (G)	9.12^*^	370.48^***^	155183^*^	116.74^***^	0.03^ns^	25.87^ns^	7.60^*^	1.79^ns^
WR x CO_2_	33.36^***^	260.37^***^	109438^*^	48.09^*^	0.03^ns^	9.69^ns^	0.77^ns^	8.16^ns^
WR x G	3.82^ns^	12.88^ns^	352201^***^	20.45^ns^	0.52^*^	50.87^ns^	3.27^ns^	13.23^*^
CO_2_ x G	16.57^***^	29.27^ns^	53189^ns^	32.64^ns^	0.03^ns^	149.20^*^	2.01^ns^	6.09^ns^
WR x CO_2_ x G	9.37^ns^	15.75^ns^	316187^**^	56.05^ns^	0.25^ns^	122.07^ns^	10.96^ns^	8.16^ns^

*SB, shoot biomass (g DW); PH, plant height (cm); SLA, specific leaf area (cm^2^/g); LC, leaf chlorophyll content (SPAD units); anthocyanin content (absorbance g^−1^ dry matter); RWC, leaf relative water content (%); Ψ_w_, leaf water potential (bar); Ψπ, leaf osmotic potential (bar).Values within each water regime are the means ± SE of 40 measurements (two levels of CO_2_ concentration, four genotypes, and five replicates per genotype), CO_2_ concentration values are the means ± SE of 60 measurements (three water regimes, four genotypes, and five replicates per genotype), while genotypic values are the means of 30 ± SE measurements (two CO_2_ concentrations, three water regimes, and five replicates per genotype). For each replicate a single plant was used. Means followed by different letters are significantly different (P < 0.05) according to Tukey's honestly significant difference (HSD) test. Analysis of variance for the same variables is shown for the water regime (WR), CO_2_ concentration (CO_2_), genotype (G), and interaction (WR x CO_2_), (WR x G), (CO_2_ x G), (WR x CO_2_ x G) effects. The associated percentage of the sum of squares and probabilities (ns, not significant*;

* *P < 0.05*;

** P < 0.01 and

*** *P < 0.001) are shown*.

The interactions between water regime (WR) and [CO_2_] were significant for all the traits of Table 1 except RWC, anthocyanin, and Ψ_w_, meaning that, except for these two traits, the response to water regime differed depending on [CO_2_]. Most of the interactions of genotypes with growing conditions were not significant, even when it is worth to mention the significant interaction between genotype and [CO_2_] for SB.

### Effect of growing conditions and genotype on gas exchange and stable isotope signatures

Water stress significantly decreased the leaf net CO_2_ assimilation (A), stomatal conductance (g_s_), the intercellular to ambient CO_2_ concentration (*C*_*i*_*/C*_*a*_), the stable oxygen isotope composition (δ^18^O) and the nitrogen concentration (N), whereas the water use efficiency (WUE), the stable carbon isotope composition (δ^13^C), and the stable nitrogen isotope composition (δ^15^N) increased and no significant differences existed for the transpiration (T) (Table 2). Rising [CO_2_] significantly increased A, g_s_, T, *C*_*i*_*/C*_*a*_, WUE, and δ^18^O, whereas N, δ^13^C and δ^15^N decreased. The genotypic effect was significant for all traits except A and T. The interaction between WR and [CO_2_] was significant for all the traits except δ^18^O (*P* = 0.060) and T. The interactions between genotype and [CO_2_] were significant for A, g_s_, *C*_*i*_*/C*_*a*_, and WUE, and near significant for T (*P* = 0.056), whereas the interaction between genotype and WR were also significant for g_s_, *C*_*i*_*/C*_*a*_, and WUE. Except for an interaction between genotype and WR for δ^15^N, no interactions between genotype and growing conditions were found.

**Table 2:** Water regime, CO_2_ concentration, and genotype effects on gas-exchange parameters, carbon, oxygen, and nitrogen isotope composition, and nitrogen concentration of four sweet pepper genotypes grown under different combinations of CO_2_ concentration and water supply.

	***A***	***g_*s*_***	***C_*i*_/C_*a*_***	***T***	**WUE**	**N (%)**	**δ^13^C (‰)**	**δ^18^O (‰)**	**δ^15^N (‰)**
**WATER REGIME**									
Control	13.03^a^ ± 0.18	314.51^a^ ± 10.71	0.79^a^ ± 0.01	4.16^a^ ± 0.39	3.34^b^ ± 0.15	6.06^a^ ± 0.06	−42.46^b^ ± 0.10	26.55^a^ ± 0.09	2.35^b^ ± 0.06
Mild stress	13.01^a^ ± 0.20	211.84^b^ ± 11.66	0.74^b^ ± 0.02	3.15^a^ ± 0.42	4.27^a^ ± 0.27	5.41^b^ ± 0.06	−41.45^a^ ± 0.10	26.21^b^ ± 0.09	2.42^ab^ ± 0.07
Moderate stress	11.68^b^ ± 0.20	169.32^c^ ± 11.48	0.69^c^ ± 0.02	3.38^a^ ± 0.41	4.50^a^ ± 0.25	4.66^c^ ± 0.06	−41.17^a^ ± 0.10	26.20^b^ ± 0.09	2.60^a^ ± 0.07
**CO_2_ CONCENTRATION**									
400 ppm	10.12 ± 0.20	222.02 ± 13.09	0.69 ± 0.01	3.11 ± 0.30	3.51 ± 0.11	5.54 ± 0.05	−35.32 ± 0.08	26.12 ± 0.07	3.12 ± 0.05
800 ppm	15.01 ± 0.21	251.56 ± 13.21	0.78 ± 0.01	4.01 ± 0.31	4.55 ± 0.22	5.27 ± 0.05	−48.03 ± 0.08	26.56 ± 0.07	1.79 ± 0.05
**GENOTYPES**									
Coyote	12.58^a^ ± 0.21	206.02^b^ ± 12.60	0.71^b^ ± 0.02	3.95^a^ ± 0.45	4.07^a^ ± 0.18	5.53^ab^ ± 0.07	−41.89^b^ ± 0.12	26.34^ab^ ± 0.11	2.67^a^ ± 0.08
Herminio	12.96^a^ ± 0.23	213.01^b^ ± 13.47	0.73^b^ ± 0.01	3.02^a^ ± 0.49	4.57^a^ ± 0.35	5.10^c^ ± 0.06	−41.74^b^ ± 0.12	26.48^a^ ± 0.10	2.43^ab^ ± 0.07
Tallante	12.27^a^ ± 0.23	280.78^a^ ± 13.54	0.77^a^ ± 0.02	3.85^a^ ± 0.49	3.38^b^ ± 0.12	5.65^a^ ± 0.07	−42.17^b^ ± 0.12	26.50^a^ ± 0.11	2.24^b^ ± 0.08
Velez	12.56^a^ ± 0.22	244.13^ab^ ± 12.52	0.73^b^ ± 0.02	3.52^a^ ± 0.45	4.06^a^ ± 0.30	5.32^bc^ ± 0.06	−41.13^a^ ± 0.11	26.03^b^ ± 0.10	2.47^ab^ ± 0.07
**LEVEL OF SIGNIFICANCE**									
Water regime (WR)	59.20^***^	389424^***^	0.16^***^	18.27^ns^	27.84^***^	32.21^***^	22.12^***^	2.82^*^	1.07^*^
CO_2_ concentration (CO_2_)	701.39^***^	22051^*^	0.20^***^	20.67^*^	30.60^***^	1.90^***^	4143.95^***^	5.02^***^	45.24^***^
Genotype (G)	0.52^ns^	73058^***^	0.05^***^	10.02^ns^	18.26^***^	4.35^***^	7.02^***^	3.75^**^	1.84^**^
WR x CO_2_	11.44^*^	36743^*^	0.03^**^	27.02^ns^	8.99^**^	10.72^***^	13.73^***^	1.71^ns^	4.86^***^
WR x G	12.84^ns^	68741^*^	0.04^*^	31.11^ns^	9.29^***^	0.13^ns^	3.20^ns^	1.81^ns^	2.44^*^
CO_2_ x G	51.42^***^	93735^***^	0.07^***^	36.72^ns^	26.51^***^	0.08^ns^	1.93^ns^	0.77^ns^	0.27^ns^
WR x CO_2_ x G	13.55^ns^	34002.^ns^	0.03^*^	27.71^ns^	12.51^*^	0.46^ns^	1.95^ns^	3.87^ns^	1.11^ns^

*A, leaf net CO_2_ assimilation (μmol CO_2_ m^−2^ s^−1^); g_s_, stomatal conductance (mmol CO_2_ m^−2^ s^−1^); C_i_/C_a_, intercellular to ambient CO_2_ concentration; T, transpiration rate (mmol H_2_O m^−2^ s^−1^); WUE, water use efficiency (μmol CO_2_ mmol H_2_O^−1^); N, nitrogen concentration (% DW); δ^13^C, stable carbon isotope composition (‰), δ^18^O stable oxygen isotope composition (‰); δ^15^N, stable nitrogen isotope composition (‰). Values within each water regime are the means ± SE of 40 measurements (two levels of CO_2_ concentration, four genotypes, and five replicates per genotype), CO_2_ concentration values are the means ± SE of 60 measurements (three water regimes, four genotypes, and five replicates per genotype), while genotypic values are the means ± SE of 30 measurements (two CO_2_ concentrations, three water regimes, and five replicates per genotype). For each replicate a single plant was used. Means followed by different letters are significantly different (P < 0.05) according to Tukey's honestly significant difference (HSD) test. Analysis of variance for the same variables is shown for the water regime (WR), CO_2_ concentration (CO_2_), genotype (G), and interaction (WR x CO_2_), (WR x G), (CO_2_ x G), (WR x CO_2_ x G) effects. The associated percentage of the sum of squares and probabilities (ns, not significant*;

* *P < 0.05*;

** P < 0.01 and

*** *P < 0.001) are shown*.

Given the significance of the interactions between WR and [CO_2_] for most of the traits in Tables 1, 2, a subset of traits in these tables was analyzed across water regimes within each [CO_2_] (Table 3). At atmospheric [CO_2_] the water regime significantly affected SB, A, g_s_, *C*_*i*_*/C*_*a*_, N, and δ^18^O, and the effect for δ^13^C (*P* = 0.068) and δ^15^N (*P* = 0.057) approached significance, whereas at high [CO_2_] the water regime significantly affected all the traits except δ^18^O. The genotypic effect at atmospheric [CO_2_] was significant for all the traits, except δ^15^N (*P* = 0.057) and SB, whereas at high [CO_2_] the genotypic effect was significant for all the traits except δ^15^N and δ^18^O. Interactions of genotypes with WR at atmospheric [CO_2_] were only significant for A, *C*_*i*_*/C*_*a*_ and δ^18^O, whereas at high [CO_2_] no interactions were detected.

**Table 3:** Effect of water regime treatments in each CO_2_ concentration (400 and 800 ppm) on shoot biomass, gas-exchange parameters, nitrogen content, and carbon, oxygen, and nitrogen stable composition.

		**SB**	**A**	***g_*s*_***	***C_*i*_/C_*a*_***	**N (%)**	**δ^13^C (‰)**	**δ^18^O (‰)**	**δ^15^N (‰)**
CO_2_ 400 ppm	Control	9.58^a^ ± 1.26	10.99^a^ ± 1.28	317.36^a^ ± 87.93	0.76^a^ ± 0.03	6.09^a^ ± 0.24	−35.61^a^ ± 0.31	26.38^a^ ± 0.41	3.12^a^ ± 0.44
	Mild stress	7.25^b^ ± 0.59	10.88^a^ ± 1.51	225.64^b^ ± 61.92	0.71^a^ ± 0.05	5.22^b^ ± 0.57	−35.13^a^ ± 0.70	25.86^b^ ± 0.25	3.27^a^ ± 0.33
	Moderate stress	6.57^b^ ± 0.56	8.65^b^ ± 1.29	126.65^c^ ± 51.86	0.62^b^ ± 0.07	5.27^b^ ± 0.31	−35.12^a^ ± 0.83	26.08^ab^ ± 0.21	2.98^a^ ± 0.26
Level of significance	Water regime (WR)	77.59^***^	49.23^***^	258979^***^	0.14^***^	7.13^***^	1.83^ns^	1.71^***^	0.74^ns^
	Genotype (G)	1.23 ^ns^	19.42^**^	39535^*^	0.04^***^	1.92^**^	4.87^**^	2.51^***^	0.098 ^ns^
	WR x G	3.21 ^ns^	19.46^*^	29874 ^ns^	0.04^**^	0.57 ^ns^	3.50 ^ns^	1.47^*^	0.47 ^ns^
CO_2_ 800 ppm	Control	11.99^a^ ± 2.06	15.68^a^ ± 1.20	312.90^a^ ± 84.00	0.82^a^ ± 0.04	6.04^a^ ± 0.25	−48.59^b^ ± 0.61	26.69^a^ ± 0.60	1.67^b^ ± 0.47
	Mild stress	10.23^b^ ± 0.92	14.86^a^ ± 1.47	217.38^b^ ± 92.96	0.77^ab^ ± 0.07	5.63^b^ ± 0.46	−48.55^b^ ± 0.65	26.68^a^ ± 0.75	1.39^b^ ± 0.48
	Moderate stress	6.23^c^ ± 1.03	14.63^a^ ± 1.22	203.60^b^ ± 77.78	0.75^b^ ± 0.08	4.14^c^ ± 0.40	−46.88^a^ ± 0.83	26.36^a^ ± 0.80	2.23^a^ ± 0.38
Level of significance	Water regime (WR)	257.65^***^	10.59^*^	110428^***^	0.03^**^	30.50^***^	30.78^***^	1.19^ns^	4.26^***^
	Genotype (G)	23.08^**^	23.67^***^	111626^***^	0.07^***^	2.53^***^	4.11^*^	1.39 ^ns^	0.59 ^ns^
	WR x G	13.96^ns^	11.36^ns^	54817^ns^	0.02 ^ns^	0.24 ^ns^	2.35 ^ns^	3.17 ^ns^	1.90 ^ns^

*Means followed by different letters are significantly different (P < 0.05) according to Tukey's honestly significant difference (HSD) test. Analysis of variance for the same variables is shown for the water regime (WR), genotype (G), and interaction (WR x G) effects. The associated percentage of the sum of squares and probabilities (ns, not significant*;

* *P < 0.05*;

** P < 0.01 and

*** *P < 0.001) are shown*.

*Values within each water regime are the means ± SE of 20 measurements (four genotypes and five replicates per genotype). For each replicate a single plant was used.SB, shoot biomass (g DW); A, leaf net CO_2_ assimilation (μmol CO_2_ m^−2^ s^−1^); g_s_, stomatal conductance (mmol CO_2_ m^−2^ s^−1^); C_i_/C_a_, intercellular to ambient CO_2_ concentration; N, nitrogen concentration (%, DW); δ^13^C, stable carbon isotope composition (‰), δ^18^O stable oxygen isotope composition (‰); δ^15^N, stable nitrogen isotope composition*.

### Relationships of shoot biomass with gas exchange and stable isotopes

The range of A rates measured across the different water conditions at the end of the experiment was only weakly correlated with SB (*r* = 0.34, *P* < 0.05) at atmospheric [CO_2_] and was not correlated at high [CO_2_] (data not shown). In fact, while A and g_s_ were less affected by water limitation at high compared to atmospheric [CO_2_], the opposite occurred for leaf growth, where the greatest decrease in biomass occurred at high [CO_2_].

**Figure 1:**
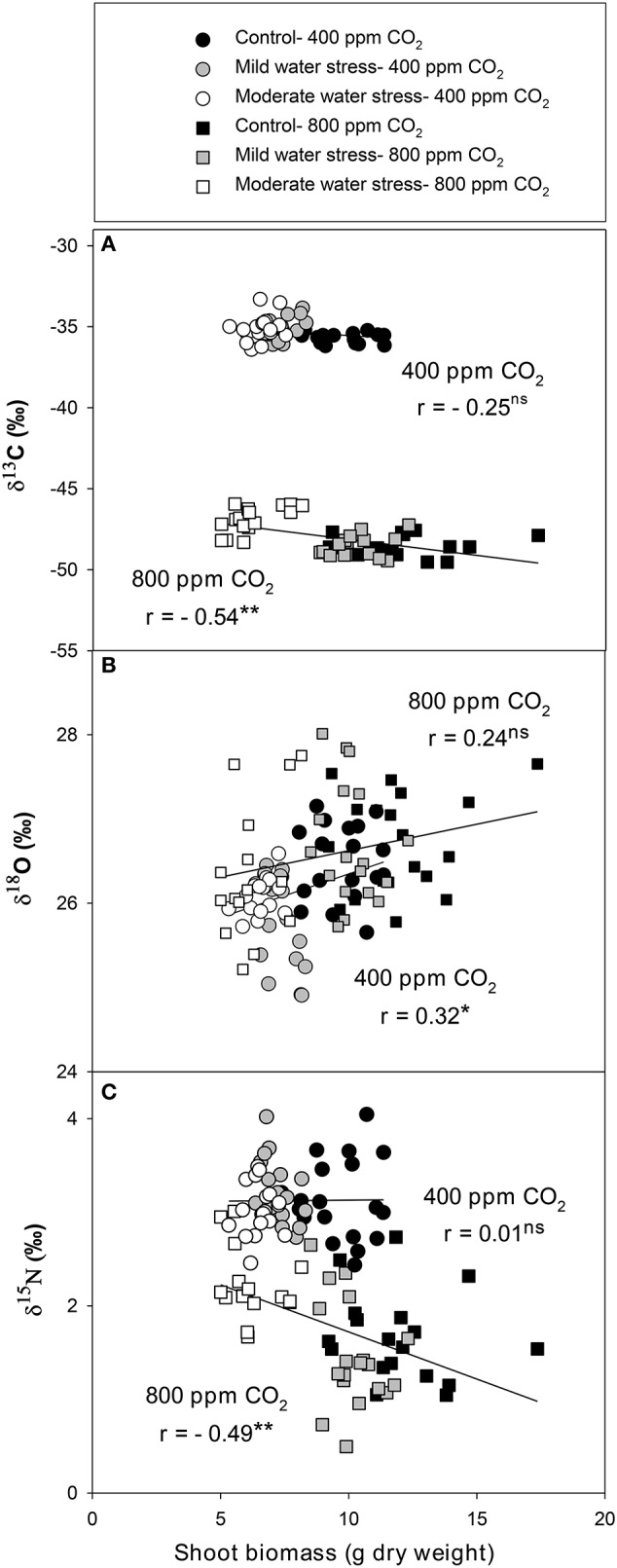
Relationships of shoot biomass with the stable isotope compositions of **(A)** carbon (δ^13^C), **(B)** oxygen (δ^18^O), and **(C)** nitrogen (δ^15^N) analyzed in the leaves of sweet pepper grown hydroponically under different [CO_2_] and water regimes. Levels of significance: ns, no significant; ^*^P < 0.05 and ^**^P < 0.01.

**Figure 2:**
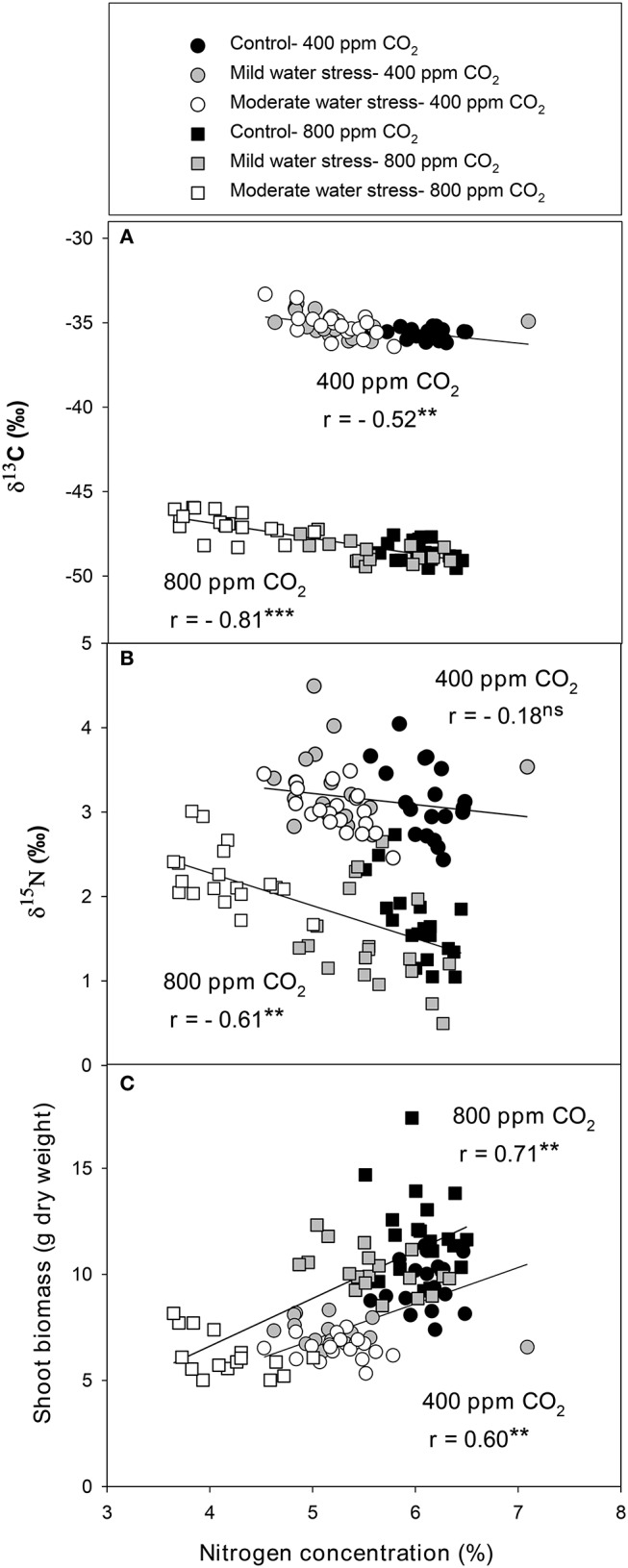
Relationships of leaf nitrogen concentration with the stable isotope compositions of **(A)** carbon (δ^13^C) and **(B)** nitrogen (δ^15^N) and the **(C)** shoot biomass of sweet pepper grown hydroponically under different [CO_2_] and water regimes. Levels of significance: ^**^P < 0.01 and ^***^P < 0.001.

The single correlations between the signatures of the different isotopes against SB were plotted for each [CO_2_] level across the three water regimes. δ^13^C correlated negatively against SB at high [CO_2_], whereas the negative relationship at atmospheric [CO_2_] did not reach significance (Figure 1A). By contrast δ^18^O correlated with SB in a weak, albeit significant, positive manner at atmospheric [CO_2_], whereas the positive relationship did not reach significance at high [CO_2_] (Figure 1B). δ^15^N correlated negatively with SB at high [CO_2_],whereas no correlation existed at atmospheric [CO_2_] (Figure 1C). The nitrogen concentration correlated positively with SB at both [CO_2_] levels (Figure 2C) in a stronger manner than any of the three stable isotopes. In addition, the N concentration correlated negatively with δ^13^C at atmospheric [CO_2_] and in a far stronger way at high [CO_2_] (Figure 2A). Moreover, δ^13^C correlated with the total shoot nitrogen content (calculated as a SB x N/100) in a weaker manner (*r* = −0.43 and −0.64, both *P* < 0.01, for 400 and 800 μmol mol^−1^ [CO_2_], respectively) than the nitrogen concentration alone (Figure 2A). In contrast the N concentration correlated negatively with δ^15^N only at high [CO_2_] (Figure 2B) and a weak positive correlation existed between nitrogen concentration and δ^18^O at atmospheric [CO_2_] (*r* = 0.33, *P* < 0.05; data not shown). Moreover, g_s_ correlated positively with the N concentration at both [CO_2_] levels, and while it also correlated with SB, this was only at atmospheric [CO_2_] (Figure S1). *C*_*i*_*/C*_*a*_ correlated with the N concentration and SB in a similar way, but in a somewhat weaker manner than g_s_.

**Figure 3:**
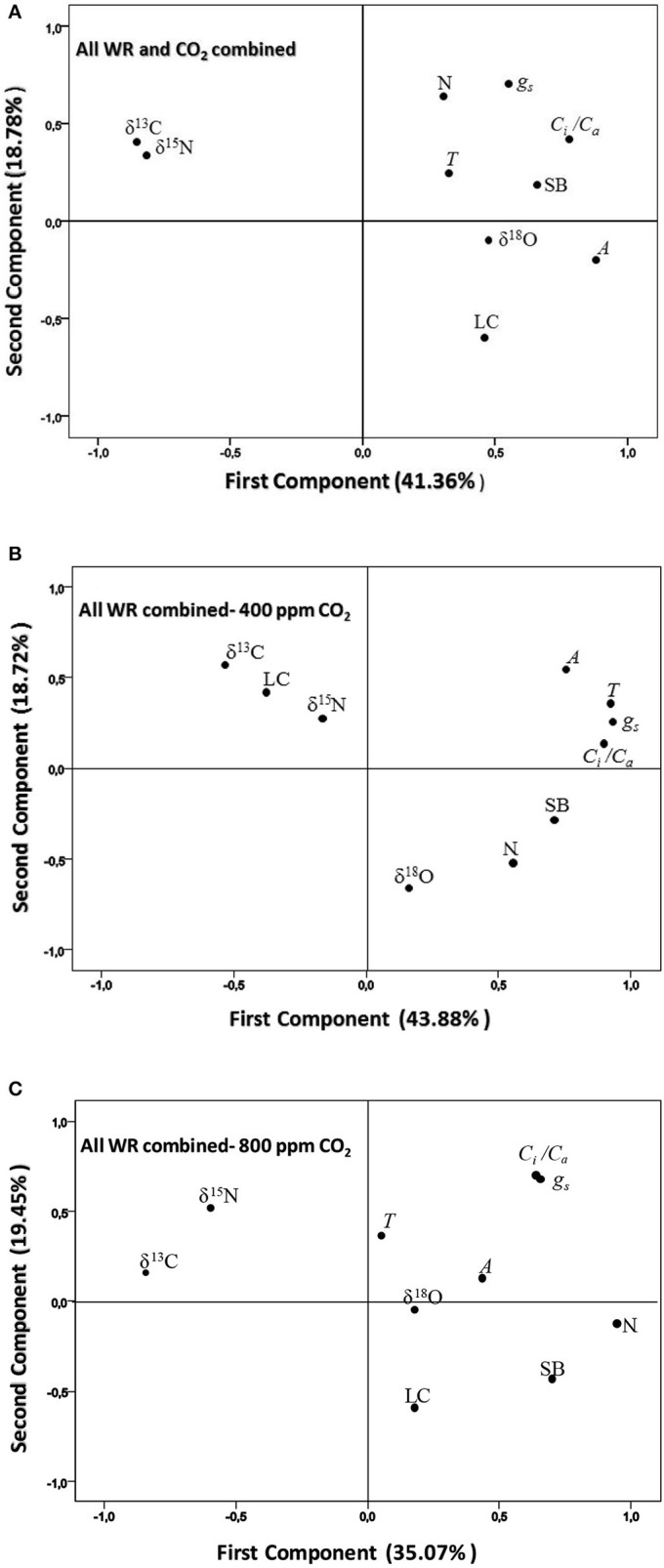
Principal component analysis of shoot biomass (SB) and different physiological traits related to the photosynthetic, transpirative, and nitrogen status of the plant were set for four sweet pepper varieties grown under **(A)** three different water regimes (WR) and two [CO_2_] combined, **(B)**, three water regimes at atmospheric [CO_2_], and **(C)** three water regimes at high [CO_2_]. The physiological traits included as variables are: the stable carbon, oxygen and nitrogen isotope compositions (δ^13^C, δ^18^ O, δ^15^N), the nitrogen concentration (N), the chlorophyll content (LC), and the net CO_2_ Assimilation (A), transpiration (T), stomatal conductance (g_s_), and the ratio of the intercellular vs. the atmospheric [CO_2_] (C_i_/C_a_) of leaves.

In order to get an overall view of the relationships between shoot biomass and all the different physiological traits, a principal component analysis (PCA) was undertaken that included SB and LC together with the gas exchange traits and stable isotope signatures in Table 2. For all the water regimes and [CO_2_] combined, the two first components explained 60% of variability. SB was placed nearly opposite to δ^13^C and δ^15^N and surrounded, at a certain distance, by δ^18^O, N and the A, g_s_, *C*_*i*_*/C*_*a*_, and T gas-exchange parameters, whereas LC was placed further away (Figure 3A). For the three water regimes at atmospheric [CO_2_] the two first component axes of the PCA accounted for more than 62% variability. In this case SB, and particularly N, were placed clearly opposite to δ^13^C, LC and δ^15^N, with the first isotope the furthest away and the second the closest to the center (Figure 3B), whereas *C*_*i*_*/C*_*a*_ (together with N) surrounded SB. The rest of the gas exchange traits were placed more (g_s_, and T) or less (A) close to *C*_*i*_*/C*_*a*_, while δ^18^O had the least alignment to SB. In the case of the PCA for the three water regimes at high [CO_2_], the two first component axes explained around 55% of variability. As in the previous PCAs, δ^13^C and δ^15^N were placed opposite to SB, whereas for the rest of the parameters only N was placed relatively near SB and all the gas exchange traits, together with δ^18^O and LC, were placed on the same side of the representation as SB but far away from it (Figure 3C).

The relationships between SB with the different traits of Table 2 in combination were assessed through a stepwise regressions analysis (Table 4). At atmospheric [CO_2_] and the three water regimes combined, the first variable chosen by the model was g_s_, which on its own explained 30% of the variability in SB. The second variable chosen by the model was N, with the two variables explaining together 44% of the variability in SB. At elevated [CO_2_] and the three water regimes combined, the first variable chosen by the model was N concentration, which on its own explained 51% of the variability in SB. The second variable chosen by the model was Ci with the two variables explaining 58% of the variability in SB. In control conditions and both [CO_2_] combined, the first variable chosen by the model was δ^13^C, explaining 34% of the variability in SB. The second variable chosen by the model was g_s_; the two variables together explaining 44% of the variability in SB. Under mild stress and both [CO_2_] combined, the first variable chosen by the model was also δ^13^C, which on its own explained 76% of the variability; the second variable chosen by the model was N concentration, with the two variables together explaining 81% of the variability in SB. Concerning the moderate stress, only T was chosen by the model and it explained merely 16% of the variability in SB.

**Table 4:** Multiple linear regressions (stepwise) explaining shoot biomass (SB) variation across genotypic groups in each CO_2_ concentration and all water regimes (WR) combined and in each water regime condition and all [CO_2_] combined as a dependent variable, and all the gas exchange traits and stables isotope signatures and nitrogen concentration in the same growing conditions as independent variables.

**Dependent variable**	**Growing condition**	**Variable chosen**	***R*^2^**	**Final stepwise model**
SB	CO_2_ 400 ppm (all WR combined)	g_s_	0.30^***^	SB = 0.006 g_s_ + 1.06 N + 0.53
		g_s_; N	0.44^***^	
SB	CO_2_ 800 ppm (all WR combined)	N	0.51^***^	SB = 2.73 N−0.02 *C_*i*_*+ 4.86
		N; *C_*i*_/C_*a*_*	0.58^***^	
SB	Control (all CO_2_ combined)	δ^13^C	0.34^***^	SB = −0.18 δ^13^C − 0.008 g_s_ + 5.49
		δ^13^C; g_s_	0.44^***^	
SB	Mild stress (all CO_2_ combined)	δ^13^C	0.76^***^	SB = −0.24 δ^13^C −0.76 N + 2.76
		δ^13^C; N	0.81^***^	
SB	Moderate stress (all CO_2_ combined)	*T*	0.16^*^	SB = −0.08 *T* + 6.67

Levels of significance:

* P < 0.05 and

*** *P < 0.001*.

*Abbreviations for variables are as defined in Table 2*.

### Path analysis

**Figure 4:**
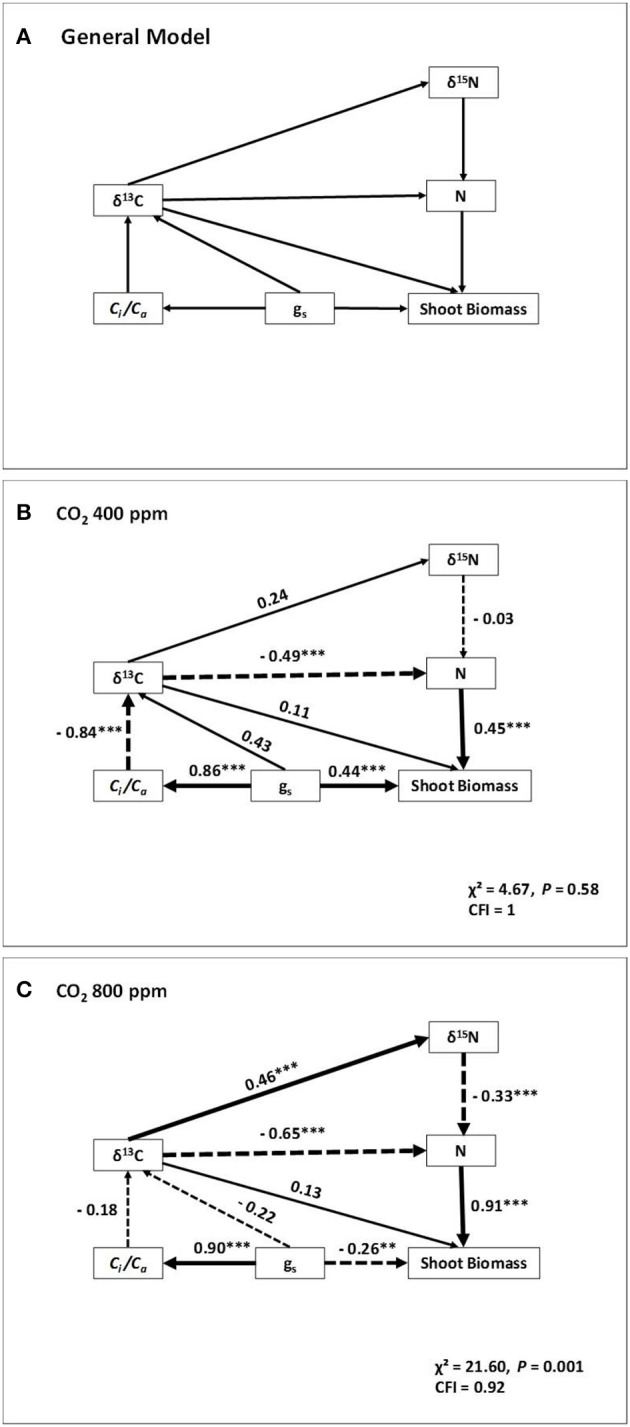
Path analyses of four sweet pepper varieties grown under different water regimes and two [CO_2_]. The conceptual model quantifying the relative strengths of direct and indirect relationships of gas exchange, stable isotope compositions and nitrogen concentration on shoot biomass (SB) is shown in **(A)**. Physiological parameters included in the model are: g_s_, stomatal conductance; C_i_/C_a_, the ratio of the intercellular vs. the atmospheric [CO_2_]; δ^13^C, carbon isotope composition; δ^15^N, nitrogen isotope composition and N concentration of leaves. The width of arrows is proportional to the path coefficient values. Dashed lines indicate negative relationships. Overall fit statistics for each path model (x^2^ and comparative fit index, CFI), the latter useful for small sample sizes (with values >0.9 taken as indicative of a good fit), are shown at the bottom right of each panel. ^**^P < 0.01, ^***^P < 0.001.

Further, we used the traits best correlated with shoot biomass to develop a conceptual model via a path-analysis. Besides SB, the model included δ^13^C and δ^15^N, (as time-integrated indicators of water conditions and nitrogen metabolism, respectively), together with the N concentration and the gas exchange traits, g_s_ and *C*_*i*_*/C*_*a*_ (Figure 4). In this case only the models across water regimes within each of the two [CO_2_] levels were assayed, but excluding the model combining the two [CO_2_], because the different δ^13^C of the ambient air and the compressed CO_2_. At atmospheric [CO_2_], both the g_s_ and N concentration had quite similar direct positive effects on the shoot biomass. However, g_s_ also indirectly affected N concentration through its strong direct effect on *C*_*i*_*/C*_*a*_ and δ^13^C. Thus, δ^13^C was negatively related with the N concentration in dry matter. The relationship of δ^13^C on δ^15^N was small, whereas there was no direct relation of δ^15^N on the N concentration. At high [CO_2_] most of the direct effect on shoot biomass corresponded to the N concentration, whereas the effect of g_s_ was minor and apparently negative. The direct effect of g_s_ on *C*_*i*_*/C*_*a*_was very strong but its direct effect on δ^13^C was minor. However, the effect of δ^13^C on N concentration was stronger than atmospheric [CO_2_] and included a direct negative effect, together with an indirect effect mediated through changes in δ^15^N.

### Amino acid profile and network analysis

**Figure 5:**
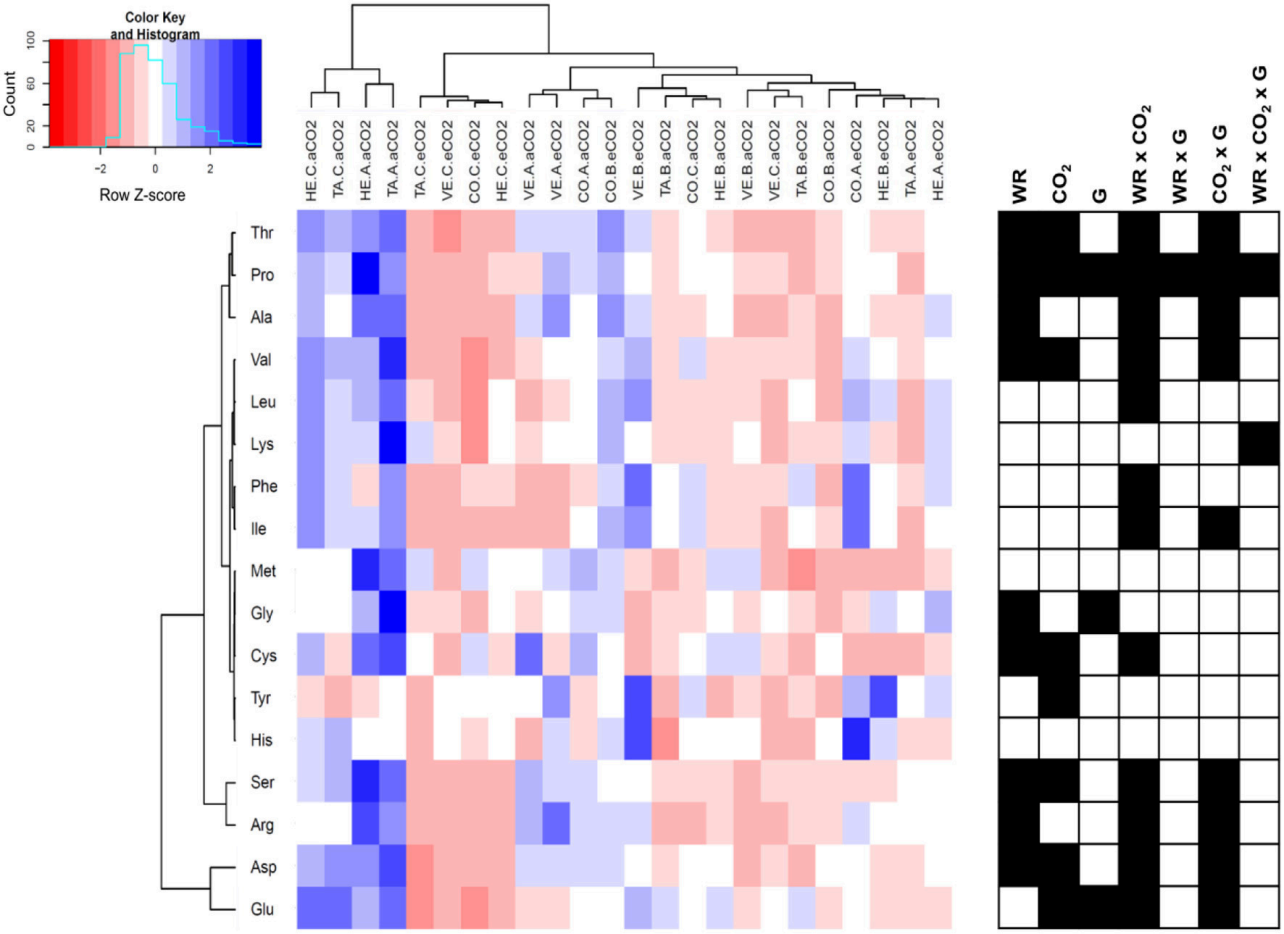
Hierarchically clustered heat map of amino acid content of four sweet pepper genotypes (TA, Tallante; CO, Coyote; HE, Herminio; VE, Velez) grown hydroponically under different [CO_2_] (aCO_2_, ambient [CO_2_]; eCO_2_, elevated [CO_2_]) and water regimes (A, control; B, mild water stress; C, moderate water stress). Values were scaled in the row direction as presented in the color key. Significance of main factors (WR, water regime; CO_2_, [CO_2_]; and G, genotype) and their interactions are shown as black cells (*P* < 0.05).

The profile of 17 amino acids were assayed in the leaves of the four sweet pepper genotypes grown under contrasting [CO_2_] and water regimes and plotted in a hierarchically clustered heat map (Figure 5). The significance of the three main factors and their interactions revealed that water regime, [CO_2_] and the CO_2_ x water stress interaction were the most relevant conditions affecting amino acid contents (all of them except lysine, methionine, and histidine). Although genotypic variability was only significant for three amino acids (proline, glycine, and glutamate), the interaction genotype by [CO_2_] affected nine amino acids, almost exactly the amino acids that were altered under the CO_2_ x water regime. CO_2_ enrichment significantly decreased the content of 8 amino acids (serine, asparagine, glutamate, threonine, proline, cysteine, and valine), and tended to decrease the content of other amino acids, and only increased the levels of tyrosine. Water stress, regardless of the severity, decreased the contents of eight amino acid (serine, glycine, asparagine, threonine, alanine, proline, cysteine, and valine). In spite of some genotypic differences the [CO_2_] x water regime interaction highlighted that, although elevated CO_2_ and water stress led to a decrease of amino acid levels, this decrease was less clear under mild compared with the most severe water stress.

Based on significant correlations between trait pairs we built a correlation network for ambient and elevated [CO_2_] treatments, including amino acid content (Figure S2). They showed similar number of nodes (32 and 31, respectively) and edges (128), although there were some differences with more negative correlations under elevated [CO_2_]. In both networks amino acid levels were positively correlated between them. However, most amino acids correlated positively with shoot biomass under elevated [CO_2_], while at ambient [CO_2_] only cysteine and tyrosine did. In ambient [CO_2_] network (Figure S2A) amino acids levels were also positively associated with SLA, N content, and δ^18^O and negatively with δ^15^N. By contrast, in elevated [CO_2_] network (Figure S2B) amino acids levels were associated with more traits: positively with PH, A, and N content and negatively with RWC, Ψ_w_, δ^13^C, T, and δ^15^N.

## Discussion

Water stress affected negatively plant growth as compared with control conditions and regardless the [CO_2_] assayed. Water stress also decreased Ψ_w_ but did not have any effect on RWC and Ψπ. These kinds of responses do not support osmotic adjustment, which contrasts with the available literature (e.g., Wullschleger and Oosterhuis, 1991; del Amor et al., 2010), and may be due to the mild to moderate nature of the water stress applied. In fact, the relatively small (but significant) changes in A rates, g_s_, LC, and δ^13^C and the lack of differences for SLA and anthocyanins under stress compared to control conditions support this statement. Moreover, although anthocyanins have protective functions during drought stress, their contribution to osmotic regulation might be low (Manetas, 2006).

The effects of high [CO_2_] on increasing plant growth and biomass have been widely reported in many plant species including pepper (del Amor et al., 2010), with the positive effect being less evident at the most severe water stress (Peñuelas et al., 1995; Medina et al., 2016). The decrease in SLA has also been reported following increases in [CO_2_] (Peñuelas et al., 1995; Piñero et al., 2016) and as a consequence of water stress (Xu et al., 2014). These results suggest that the leaf thickness of mesophyll packing increased in response to high [CO_2_] (Oberbauer et al., 1985). The LC and anthocyanins also increased in response to high [CO_2_]. Previous results in pepper only exhibited a trend toward higher chlorophyll content following exposure to high [CO_2_] (Peñuelas et al., 1995). The increase of anthocyanins under elevated [CO_2_], as it was also observed in Takatani et al. (2014) in response to higher carbon/nitrogen balance observed under these growth conditions. Indeed, the accumulation of anthocyanins is an indicative of nitrogen limitation in the plant according to these authors. The high sucrose levels usually reported under elevated [CO_2_] might stimulate the expression of MYB75/PAP1 transcription factor that further enhances the production of anthocyanins (Tzin and Galili, 2010). Moreover, the levels of tyrosine, a precursor of anthocyanins, were induced under elevated [CO_2_] and they could also induce the anthocyanin biosynthesis, as showed in *Arabidopsis thaliana* (Zhou et al., 2014). Regarding the genotype x water regime interaction, we observed that in most of the genotypes anthocyanin content increased with water stress as a response to water limitation. However, in the case of Coyote we observed a reduction of anthocyanins with water stress; that could suggest a limitation of the protective functions of anthocyanins in response to water stress in this genotype, which in part accounts for the lowest shoot biomass of this genotype.

In addition, high [CO_2_] decreased leaf RWC and Ψ_w_, and increased Ψπ. A previous study in pepper found a tendency to lower Ψ_w_ in response to high [CO_2_] under well-watered conditions, whereas the RWC increased regardless of the water regime considered, and the Ψπ increased but only under water stress (del Amor et al., 2010). However, the high [CO_2_] concentration assayed in this paper was nearly three times higher than in the present study. The effect of high [CO_2_] decreasing water status seems fairly surprising, but it may be due to a larger transpiration area compared with plants grown under normal [CO_2_].

As expected high [CO_2_] increased A and WUE compared with atmospheric [CO_2_], but surprisingly did not decrease g_s_ and transpiration under control and mild-stress conditions, and even these rates increased under moderate water stress. A small number of studies have noted similar patterns, including work on sweet pepper (del Amor et al., 2010; del Amor, 2013), other herbaceous crops (Morison, 1998; Medina et al., 2016) and in tree species that were not acclimated (Medlyn et al., 2001). The results of the current study suggest that there was no acclimation of g_s_ to elevated [CO_2_]. Moreover, the response of photosynthesis to growth in elevated [CO_2_] is commonly tested by comparing the gas exchange of plants grown at atmospheric- and high-[CO_2_] at the same [CO_2_] across both sets of plants (e.g., Drake et al., 1997; Curtis and Wang, 1998).

### Effect of [CO_2_] and water regime on δ^13^C, δ^18^O, and δ^15^N

The increase in δ^13^C under water stress is in line with earlier work (Farquhar et al., 1982, 1989). However, the effect was more evident at high [CO_2_], which agrees with the findings of del Amor (2013) for sweet pepper plants that were subjected to different levels of salinity under these two different [CO_2_]. This may be due to the δ^13^C dilution effect of the CO_2_ used to raise the air [CO_2_] up to 800 ppm (i.e., the industrial CO_2_ is a by-product of combusting fossil fuels and therefore its δ^13^C is far more negative than atmospheric CO_2_). Indeed, δ^13^C decreased around 3‰ for each 100 μmol mol^−1^ of increase in [CO_2_], which is in line with previous reports (Schubert and Jahren, 2012; del Amor, 2013). Therefore at high [CO_2_], water stress slows the increase of new biomass with lower δ^13^C. The effect (pointed out above) of high [CO_2_] decreasing water status due to a larger transpiration area, compared with plants grown under normal [CO_2_], may be also involved in the larger range of δ^13^C values as response to water stress. Thus, at high [CO_2_] the absolute changes in δ^13^C were more in parallel with the SB than with the net assimilation or *C*_*i*_*/C*_*a*_. In that regard, δ^13^C correlated negatively with SB across water regimes at high [CO_2_], but the negative relationship did not reach significance at atmospheric [CO_2_]. Significantly, stronger negative relationships between δ^13^C and SB at high vs. atmospheric [CO_2_] have been reported before for sweet pepper when plants growing across different levels of salinity were compared (del Amor, 2013). Moreover, genotypic effects on δ^13^C were significant at both [CO_2_]. To the best of our knowledge there are no studies reporting on the genotypic variability in δ^13^C of sweet pepper under different levels of water stress and [CO_2_].

Water stress slightly decreased δ^18^O but only at high [CO_2_]. Such decreases in δ^18^O in response to water stress and a lower transpiration are opposite to most of the reports (Barbour and Farquhar, 2000; Farquhar et al., 2007; Cabrera-Bosquet et al., 2009a, 2011). The increase in δ^18^O in plant material exposed to high [CO_2_] has been reported before and may be caused by increases in the δ^18^O of the leaf water (Cooper and Norby, 1994). Although it has been shown that the oxygen isotopic content of atmospheric carbon dioxide has little direct influence on the δ^18^O of either leaf water or cellulose (DeNiro and Epstein, 1979), increasing atmospheric carbon dioxide concentrations may have significant indirect effects on heavy stable isotope enrichment in leaf water. This would result from the expected decreases in transpiration and increases in WUE by plants as [CO_2_] levels increase (Eamus and Jarvis, 1989). The processes leading to concentration of the heavy isotopes ^18^O in leaf water are similar in many respects to evaporation, which alters the isotopic composition of terrestrial surface waters (Cooper and Norby, 1994). Alternative explanations for the increase in δ^18^O at high [CO_2_] are not supported by our results. Indeed, a higher CO_2_ release due to photorespiration at atmospheric compared with high [CO_2_] levels would cause an increase in δ^18^O at atmospheric [CO_2_] levels (Farquhar et al., 1993), which does not agree with the increase we found in δ^18^O at high [CO_2_]. Changes in g_s_ do not seem to be involved because, regardless of the presence or absence of differences in g_s_ within a given water regime between the atmospheric and high [CO_2_], all water regimes at high [CO_2_] exhibited higher δ^18^O than the corresponding water regimes at atmospheric [CO_2_]. Moreover, genotypic variability was only significant at atmospheric [CO_2_]. In spite of some positive results (Barbour et al., 2000; Cabrera-Bosquet et al., 2009b), the weak performance of δ^18^O in correlating with biomass and yield has been extensively reported (Araus et al., 2013; Bort et al., 2014; Foulkes et al., 2016; Munjonji et al., 2016). This poor performance appears to be caused by post photosynthetic fractionations of the ^18^O signature in the photoassimilates (Sánchez-Bragado et al., 2016). The same reasoning may be extended for the weak and erratic correlations of δ^18^O with gas exchange traits and δ^13^C that we found.

Plants under the strongest water stress exhibited slightly lower δ^15^N values than the other two treatments at atmospheric [CO_2_], and the opposite occurred at high [CO_2_]. Decreases in δ^15^N following water limitation (Araus et al., 2013; Bort et al., 2014) or growing conditions causing water stress, such as salinity (Yousfi et al., 2009, 2012), have been reported before. The effect of rising [CO_2_] on δ^15^N was clearer than the effect of the water regime; in this case decreasing the isotopic composition, irrespective of the water regime considered. Depletion of the heavier N isotope in plants grown under high [CO_2_] and water deficit conditions has been reported before in a study with alfalfa (Ariz et al., 2015), whereas another recent study, this time in durum wheat, concluded that elevated [CO_2_] was the main factor that increased δ^15^N (Medina et al., 2016). The decrease in δ^15^N under elevated [CO_2_] may reflect decreased g_s_, but could also be related to a higher nitrogen demand in leaves, as suggested by the decreased in leaf N (Ariz et al., 2015) or shifts in nitrogen metabolism associated with decreases in photorespiration (Tcherkez, 2010).

The photorespiratory nitrogen cycle extending over different plant compartments implicates several reactions related to nitrogen recycling that may have ^15^N-kinetic isotope effects (Yu and Woo, 1991; Werner and Schmidt, 2002). Normally, the kinetic isotope effects on these reactions should not become evident when there is a total recycling of the intermediates without input or net production of substrates and products (Werner and Schmidt, 2002). However, plants can excrete gaseous ammonia (Francis et al., 1997; Pearson et al., 1998) as a consequence of photorespiration. If the uptake/loss of NH_3_ from plant stomata is rate-limited by the diffusion of NH_3_ in air, the transported NH_3_ will be depleted in ^15^N by around 18‰ relative to the δ^15^N of the source (Farquhar et al., 1980; Tcherkez and Hodges, 2008). As a consequence an increase in δ^15^N may be expected as a response to photorespiration, which may be the case for plants exposed to atmospheric [CO_2_] compared to enhance [CO_2_].

The correlation of δ^15^N with SB across water regimes was only significant (and negative) at the high [CO_2_]. The lack of correlation at atmospheric [CO_2_] may be due, as for δ^13^C, to the relatively narrow range of variability in SB associated with the response to water regimes at atmospheric [CO_2_]. Negative relationships between δ^15^N and SB and yield at atmospheric [CO_2_] have been frequently reported (Robinson et al., 2000; Yousfi et al., 2009; Raimanová and Haberle, 2010; Araus et al., 2013).

### Growing conditions, N concentration, and plant growth

The effects of water stress decreasing the N concentration in leaves have been extensively reported (Shangguan et al., 2000; Yousfi et al., 2012). In accordance with this, the N concentration was positively correlated with the g_s_ (*r* = 0.36, *P* < 0.01; *r* = 0.51, *P* < 0.001, at atmospheric and high [CO_2_], respectively), suggesting that the N concentration in leaves depends to some extent on the transpirative stream (Dalla Costa and Gianquinto, 2002). This may be the case for plants growing under hydroponic conditions in particular, where water and nitrogen are provided together through the nutrient solution (Peñuelas et al., 1995) and the transpiration stream largely determines the availability of mineral N in the rhizosphere (Gonzalez-Dugo et al., 2010). In support of that, our results show a general decrease in the content of the different amino acids as response to water stress. Such decrease is nonspecific, as shown by the fact these amino acids belong to different metabolic pathways (Galili et al., 2016). This may highlight that primary substrate(s) for the synthesis of all amino acids were reduced, involving the provision of C skeletons or N. It has been reported for Arabidopsis plants that a reduction in the levels of transpiration, decreased the capacity for nitrogen uptake and the shoot nitrogen concentration of the plant but only when water availability was not restricted (Hepworth et al., 2015).

Increasing the [CO_2_] had a significant, albeit minor effect, decreasing the N concentration. In fact, the results of an increase in [CO_2_] decreasing N concentration were only significant at the strongest water stress. A decrease in nitrogen concentration has been widely reported in non-leguminous plants following increases in [CO_2_] (Jablonski et al., 2002), irrespective of the water regime (Medina et al., 2016). In fact, the predicted growth response to elevated [CO_2_] is reduced at low N availability (McMurtrie et al., 2008; Vicente et al., 2016b). In our study, water deficit combined with high [CO_2_] caused the lowest SB and N concentration in the leaves among the six different growing conditions.

As expected at elevated [CO_2_], the levels of photorespiratory intermediates, glycine and serine decreased (Geiger et al., 1998, 1999; Yu et al., 2012; Aranjuelo et al., 2013; Noguchi et al., 2015). However, different to previous studies (Fritz et al., 2006; Krapp et al., 2011; Noguchi et al., 2015), the levels of (other) major amino acids (e.g., aspartate, asparagine, glutamate, and alanine) decreased. Such decrease has been reported as response to insufficient nitrogen conditions; specifically under ${\text{NO}}_{3}^{-}$ and ambient [CO_2_] conditions (Noguchi et al., 2015; Vicente et al., 2016b). This pattern suggests that in our system some limitation of N availability may be present. Nevertheless, the relative decrease of glycine as response to inhibition of photorespiration (i.e., at high compared with ambient [CO_2_]) was much lower than that of serine which is against a low N availability (Sulpice et al., 2013; Noguchi et al., 2015). On the other hand in agreement with previous studies the amount of minor amino acids (e.g., threonine, valine, cysteine, methionine, lysine, leucine, etc.) decrease at elevated [CO_2_] (Noguchi et al., 2015).

Summarizing the decrease in the amount of several amino acids under elevated CO_2_, reported in our study may have several causes. Some of them, decreased probably due to the inhibition of photorespiration under elevated CO_2_ (glycine and serine). The rest of the amino acids could have been reduced due to the inhibition of N assimilation; this has been reported in several plants under elevated CO_2_, such as Arabidopsis and wheat (Aranjuelo et al., 2013; Noguchi et al., 2015; Vicente et al., 2016b). The inhibition of N assimilation under elevated CO_2_ is not completely understood, but it could be due, as reported in Vicente et al. (2016b) and in agreement with previous studies (Bloom et al., 2010; Aranjuelo et al., 2013), to (i) lower rates of photorespiration in elevated CO_2_ that decrease the availability of reductant in the cytosol, and inhibition of ${\text{NO}}_{2}^{-}$ influx into chloroplasts; or to increased demand for ${\text{NO}}_{3}^{-}$ to match the increase in net photosynthetic assimilation in elevated CO_2_, leading to a decline of the metabolic ${\text{NO}}_{3}^{-}$ pool that restricts induction of nitrate reductase and thus ${\text{NO}}_{3}^{-}$ assimilation.

Interestingly tyrosine was the only amino acid clearly increasing as response to a high [CO_2_]. Moreover, under water stress, high [CO_2_] also increased phenylalanine. Both are aromatic amino acids, which have low N/C ratios, involved as precursors in the synthesis of anthocyanin. In fact an increase in both amino acids has been reported in Arabidopsis leaves at elevated [CO_2_] in ${\text{NH}}_{4}^{+}$ + ${\text{NO}}_{3}^{-}$ media (Noguchi et al., 2015) which is our case (i.e., nutrient solution). Cysteine was strongly decreased under elevated [CO_2_] regardless of water regime. It constitutes the first stable product of the sulfur assimilation, and acts as precursor of the majority of organic sulfur compounds (Hawkesford et al., 2012).

Independently of [CO_2_], the levels of all amino acids were correlated (Supplementary Material Figure S2). However, the relationships between amino acids with other physiological traits varied depending on [CO_2_]. It was especially remarkable that amino acid contents correlated positively with SB under high [CO_2_] and negatively with traits related to water status (RWC, Ψ_w_, δ^13^C, and T), while under atmospheric [CO_2_] the correlations between amino acids and other traits were scarce. These findings suggest that amino acid pool greatly influences biomass accumulation under elevated [CO_2_] and its amount is influenced by leaf and plant water status in a highly-dependent manner.

Decreases in transpiration associated with mass flow of soil solution, have been proposed to limit plant N acquisition. In an experiment with cottonwood, where relative humidity and atmospheric [CO_2_] were manipulated to alter the transpirative stream, N gain was positively correlated across all treatments with root mass, and a significant portion of the remaining variation (44%) was positively related to transpiration per unit root mass (McDonald et al., 2002). Thus, decreases in plant N concentration under water stress are attributable in part to associated decreases in g_s_ and transpiration. However, other mechanisms may be involved for the decrease in plant N under [CO_2_] enrichment. Thus, in our study no clear differences existed in the rates of g_s_ measured at different [CO_2_] and even transpiration was increased at high [CO_2_] relative to atmospheric [CO_2_]. Besides the potential effect of diminishing transpirative stream, [CO_2_] enrichment is reported to inhibit the assimilation of nitrate into organic nitrogen compounds (Bloom et al., 2010; Vicente et al., 2016b). This inhibition may be largely responsible for [CO_2_] acclimation, that is, the decrease in photosynthesis and growth of C_3_ plants after long exposures to [CO_2_] enrichment. Different studies have shown that the effect of elevated [CO_2_] on reducing N content was related at the transcript level to a down-regulation of genes encoding for Rubisco subunits and N-assimilation enzymes (GS1 and GS2), indicating a co-regulation of primary C and N metabolism (Stitt and Krapp, 1999; Vicente et al., 2015, 2016a,b; Medina et al., 2016).

As a consequence of the effect of water regime on N concentration, the trait best correlated with total biomass within each of the two [CO_2_] was the total leaf N concentration (and amino acid contents), although the correlation was stronger at high [CO_2_]. Overall, the results showed that greater plant growth across water regimes was linked to an increase in shoot N concentration associated to a higher transpiration stream, even when changes in N metabolism appear also involved (Hirel et al., 2007; Medina et al., 2016). A study of Peñuelas et al. (1995) on sweet pepper also found that the effect of [CO_2_] and water regime was dependent on the accumulated N supply. All these studies have plants grown under hydroponic conditions in common, where irrigation and fertilization are provided together through the nutrient solution under relatively low photosynthetic photon flux density.

The leaf N concentration was negatively correlated with δ^13^C within each [CO_2_] across water regimes, whereas nitrogen concentration and g_s_ correlated positively (but in a weaker manner). Moreover, δ^13^C correlated with the total shoot nitrogen content in a weaker manner than the nitrogen concentration alone. In fact, both the δ^13^C and nitrogen concentration were expressed on a dry matter basis, which may explain their better correlation. δ^13^C is not just an indicator of water use efficiency (Farquhar et al., 1982, 1989) but it is also strongly negatively affected by the amount of available water (Araus et al., 1997, 2003) and therefore when analyzed on a dry matter basis it may be considered an indicator on the total (i.e., through time) water used by the plant. Moreover, in the Principal Component Analysis the nitrogen concentration was placed clearly opposite to δ^13^C. In other words, the negative relationship between δ^13^C and nitrogen concentration on a dry leaf basis may be understood as the nitrogen concentration in the leaves being, at least in part, the consequence of the amount of water transpired by the plant. The Path Analysis further supported the direct contribution of the nitrogen concentration in determining total biomass as well as the positive role of an increased water use (assessed through a lower δ^13^C of the dry matter) on the N accumulation. This model worked better under high [CO_2_]. In sweet pepper growing under hydroponic conditions, strong negative correlations across salinity levels for leaf δ^13^C with both g_s_ and nitrate accumulation have been reported, with the correlations being higher at elevated [CO_2_] compared with atmospheric [CO_2_] (del Amor, 2013).

## Conclusion

The signatures of the three different stable isotopes are significantly affected by water regime, [CO_2_], and genotypic effects. However, the results do not support the use of δ^18^O as an indicator of the effect of growing conditions and genotypes on plant growth. This study proves that the effect of water regime on sweet pepper growth in a hydroponic system is caused by changes in the amount of nitrogen assimilated, which is associated with the plant's water use. In that sense, the role of δ^13^C in explaining differences in plant growth across water regimes appears mediated via its direct relationship with N accumulation in leaves, particularly at high [CO_2_]. However, our study does not support stomatal closure as being associated with an elevated [CO_2_]-induced reduction in N concentration in the shoot.

## Author contributions

FdA and JA conceived and designed the experiments. MP, MS, and GO-A contributed to the experimental work. SY and RV contributed to the data analysis and preparation of tables and figures. MS wrote the paper under the supervision of FdA and JA and all three revised the manuscript. All authors read and approved the final manuscript.

### Conflict of interest statement

The authors declare that the research was conducted in the absence of any commercial or financial relationships that could be construed as a potential conflict of interest.
