# Supplementary material for: Interactive Effects of CO2 Concentration and Water Regime on Stable Isotope Signatures, Nitrogen Assimilation and Growth in Sweet Pepper
Source: Front Plant Sci. 2018 Jan 4;8:2180. doi: 10.3389/fpls.2017.02180 (PMC5758588; doi:10.3389/fpls.2017.02180)
Supplement: Supplementary file 1 [file DataSheet1.PDF]

# Supplementary material

## Figure S1

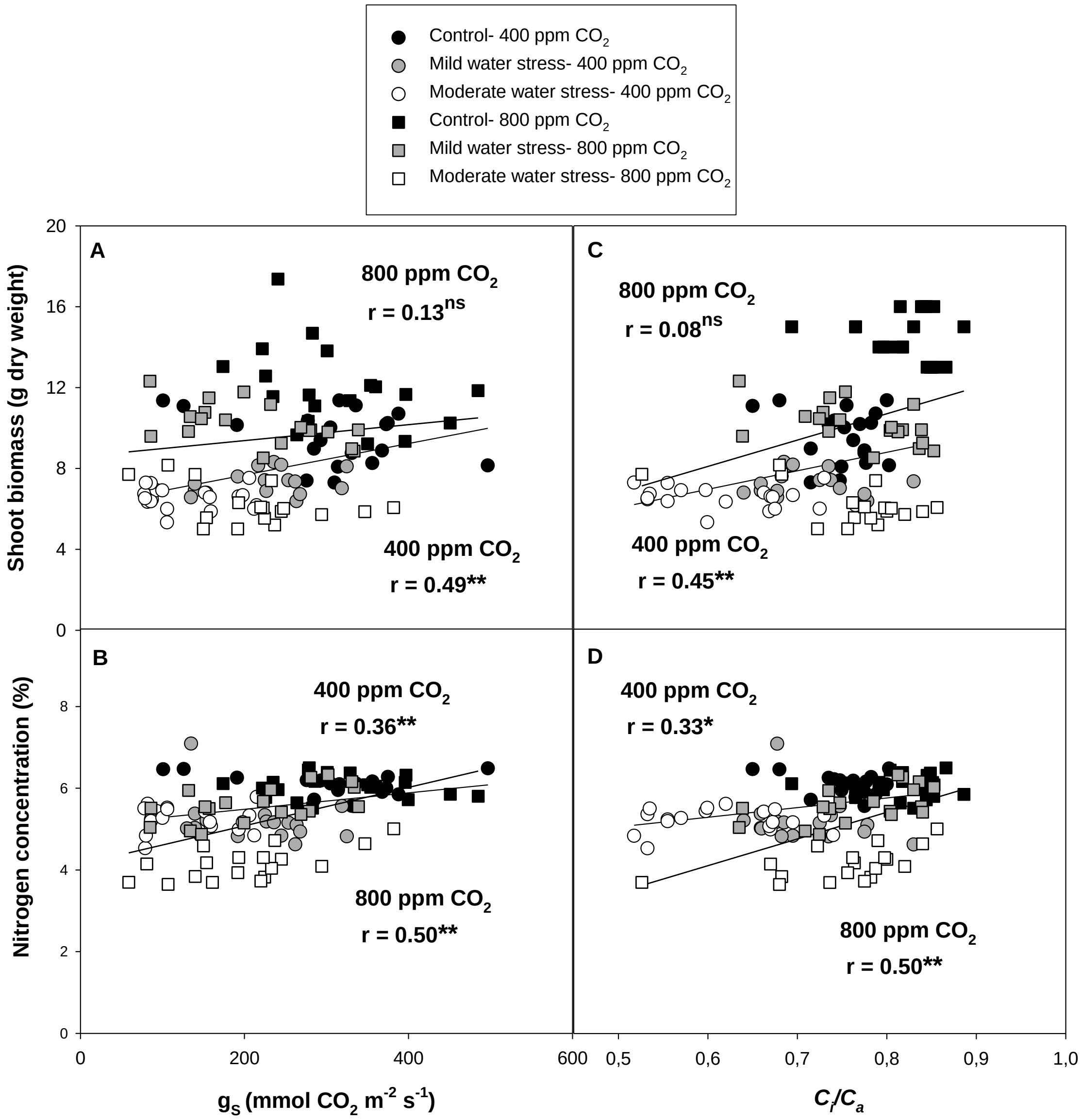


**Figure S1.** Relationships of stomatal conductance ($g_s$) and the ratio of the intercellular versus the atmospheric [$CO_2$] ($C_i/C_a$) with the shoot biomass (A, C) and the leaf nitrogen concentration (B, D) of sweet pepper grown hydroponically under different [$CO_2$] and water regimes. Levels of significance: ns, no significant; *P < 0.05 and **P < 0.01.

**Figure S2**

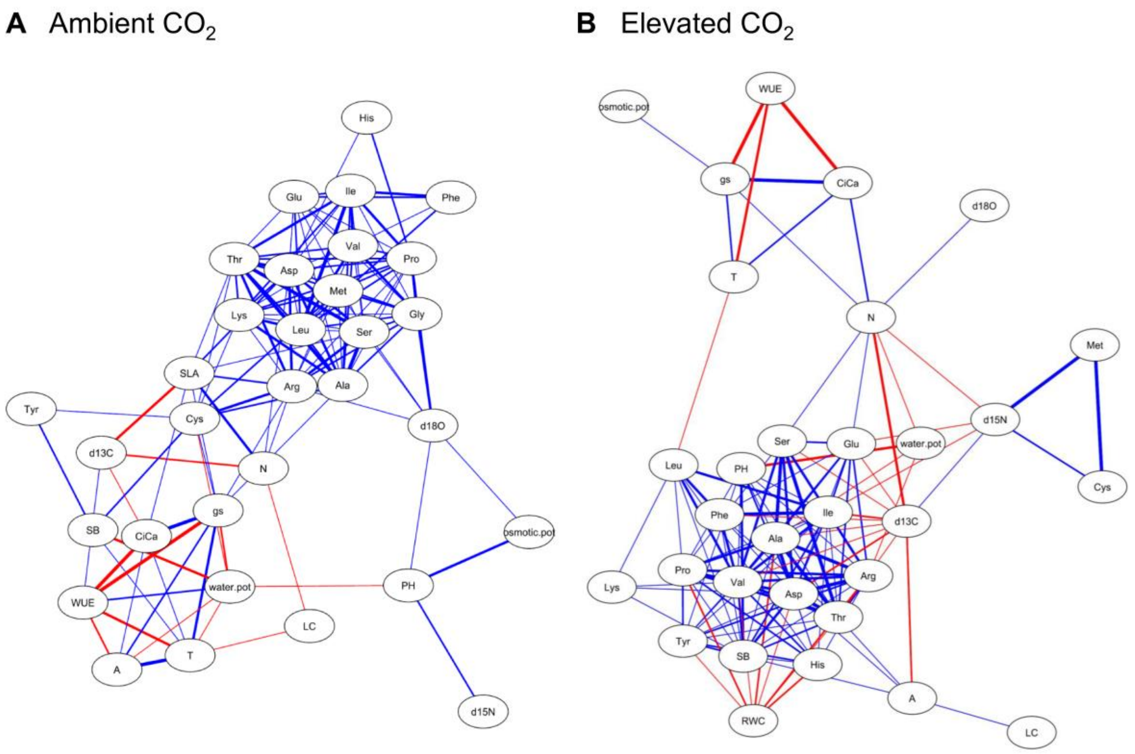


**Figure S2.** Network analysis of significant correlations between trait pairs in sweet pepper grown hydroponically under different [CO2]. Blue and red edges indicate positive and negative correlations based on Pearson correlation coefficient, respectively.
